# High efficacy of artemether-lumefantrine and declining efficacy of artesunate + sulfadoxine-pyrimethamine against *Plasmodium falciparum* in Sudan (2010–2015): evidence from in vivo and molecular marker studies

**DOI:** 10.1186/s12936-016-1339-x

**Published:** 2016-05-21

**Authors:** Ahmed A. Adeel, Fahad Awad Ali Elnour, Khalid Abdalmutalab Elmardi, Mona B. Abd-Elmajid, Mai Mahmoud Elhelo, Mousab S. Ali, Mariam A. Adam, Hoda Atta, Ghasem Zamani, Marian Warsame, Amy Barrette, Hanan El Mohammady, Rania A. Nada

**Affiliations:** College of Medicine, King Saud University, Riyadh, Saudi Arabia; National Malaria Control Programme, Federal Ministry of Health, Khartoum, Sudan; Malaria Control and Elimination, Division of Communicable Diseases Control, World Health Organization Regional Office for the Eastern Mediterranean, Cairo, Egypt; Global Malaria Programme, World Health Organization, Geneva, Switzerland; Naval Medical Research Unit-3, Cairo, Egypt

**Keywords:** Sudan, Sulfadoxine-pyrimethamine, Artesunate, Artemether-lumefantrine antimalarial drugs, Drug resistance, Molecular markers, *dhfr*, *dhps*

## Abstract

**Background:**

The present paper reports on studies that evaluated artesunate + sulfadoxine-pyrimethamine (AS + SP) which is the first-line drug and artemether-lumefantrine (AL) which is a second-line drug against uncomplicated falciparum malaria in Sudan. This evaluation was performed in twenty studies covering six sentinel sites during five successive annual malaria transmission seasons from 2010 to 2015.

**Methods:**

The standard World Health Organization protocol was used for a follow-up period of 28 days. The frequency distribution of molecular markers for antifolate resistance in dihydrofolate reductase (*dhfr*) and dihydropteroate synthase (*dhps*) genes was studied in pre-treatment samples in four sites in 2011.

**Results:**

In the nine studies of AL conducted at five sites (n = 595), high PCR-corrected cure rates were found, ranging from 96.8 to 100 %. Among the eleven studies of AS + SP (n = 1013), a decline in the PCR-corrected cure rates was observed in Gedaref in Eastern Sudan: 91.0 % in the 2011–12 season and 86.5 % in the 2014–15 season. In the remaining sites, the AS + SP cure rates ranged between 95.6 and 100 %. The rate of clearance of microscopic gametocytaemia after treatment was not significantly different with AL or AS + SP on days 7, 14, 21 and 28 of follow-up. A total of 371 pre-treatment samples were analysed for molecular markers of SP resistance. The temporal changes and geographical differences in the frequency distribution of SP-resistance genotypes showed evidence of regional differentiation and selection of resistant strains.

**Conclusion:**

The findings of this study call for a need to review the Sudan malaria treatment policy. Epidemiological factors could play a major role in the emergence of drug-resistant malaria in eastern Sudan.

**Australian New Zealand Clinical Trials Registry:**

*Trial registration numbers* 2011–2012: ACTRN12611001253998, 2013–2015: ACTRN12613000945729

## Background

Malaria in Sudan is mainly caused by *Plasmodium falciparum* (95 % of cases), followed by *P. vivax* (5 %). Among the population of 37.9 million, 87 % live in areas of high malaria transmission, and the remaining live in areas of low transmission [1]. By 2004, the prevalence of chloroquine-resistant falciparum malaria was excessively high, leading the National Malaria Control Programme (NMCP) to change its malaria treatment policy. Chloroquine was replaced by artesunate + sulfadoxine-pyrimethamine (AS + SP) as the first-line treatment policy for uncomplicated falciparum malaria and artemether-lumefantrine (AL) was adopted as a second-line treatment [2].

Around the time when AS + SP was adopted as the first-line treatment for falciparum malaria, a number of therapeutic efficacy studies evaluated SP in Sudan, either as a monotherapy or in combination with other anti-malarials [3]. These studies reported variable rates of treatment failure with SP monotherapy, reaching 31.7 % in eastern Sudan in 2003 [4]. Therapeutic efficacy studies (TES) for anti-malarial drugs are the gold standard for guiding anti-malarial treatment policy. The World Health Organization (WHO) recommends that studies of first- and second-line anti-malarial medicines should be conducted once every 2 years at sentinel sites within each country [5]. Molecular markers for SP resistance provide supporting evidence and can predict resistance to SP to a reasonable extent [6]. These markers could provide a very convenient and cost effective tool to monitor changes in the prevalence of drug-resistant parasites. An increasing prevalence of alleles of genes known to confer resistance to a drug can provide an early warning of developing resistance [7]. Equally, a decrease in resistant alleles can be an indication of returning sensitivity when a drug is withdrawn [8, 9]. Molecular marker surveys could also greatly extend the coverage of resistance monitoring in areas where in vivo tests are not feasible, even though they do not replace in vivo tests. This paper reports the results of therapeutic efficacy studies on AL and AS + SP conducted in Sudan from 2010 to 2015. An analysis of molecular markers conferring resistance to SP resistance is provided for four sentinel sites studied in 2011.

## Methods

### Study sites

**Fig. 1:**
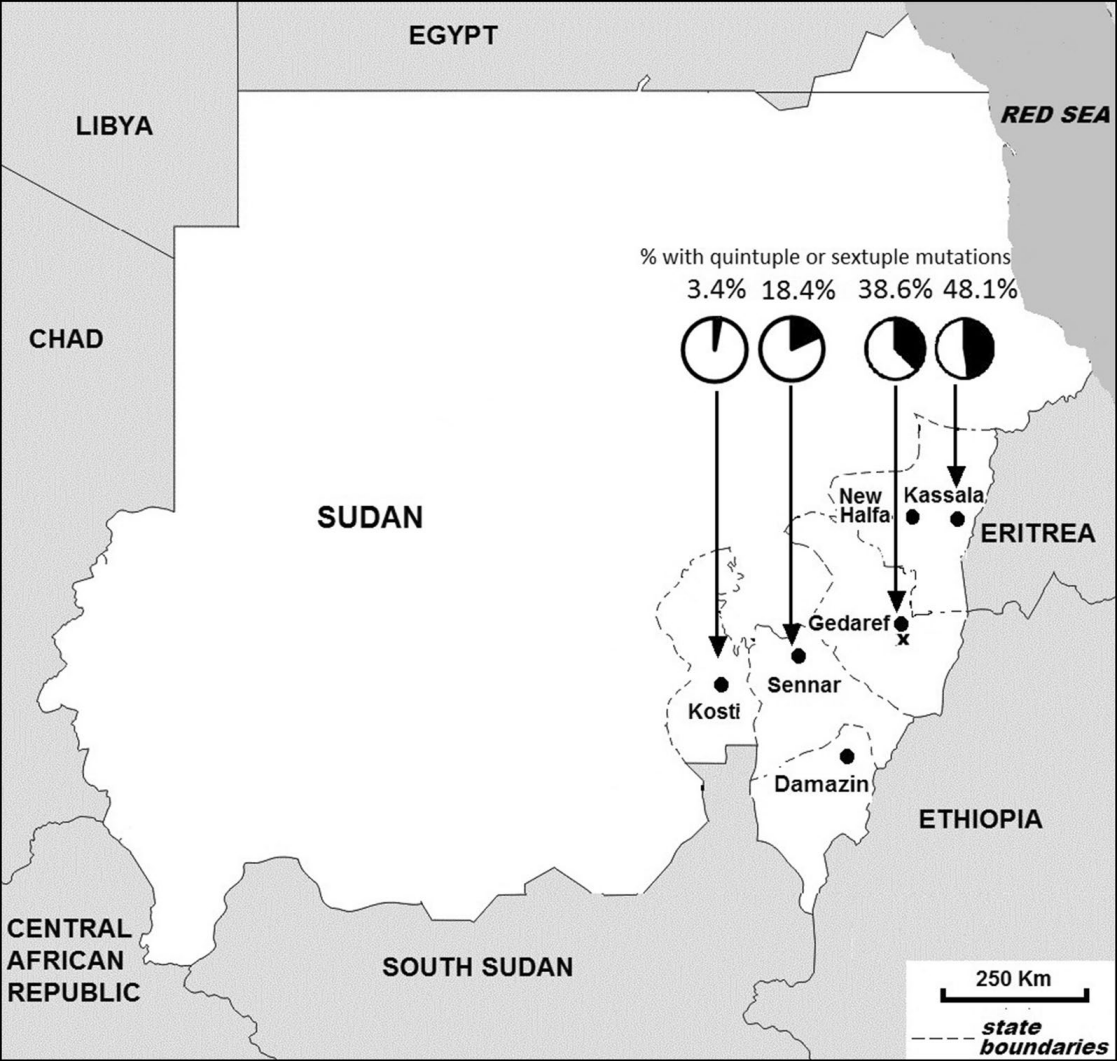
Map of Sudan showing six sentinel sites for therapeutic efficacy tests in Sudan 2010–2015. “X” indicates the location of three villages where isolates were collected in previous SP molecular marker studies in 2003. *Pie charts* indicate the proportion with quintuple/sextuple mutations. *Black* segments indicate the percentage of samples with quintuple or sextuple mutations in each site

Figure 1 shows the six sentinel sites and the states covered. The transmission season was from July/August to November/December, with an earlier start in June in the southern areas (e.g., Damazin) and a later start in August in northern areas (e.g., Kassala). Longer transmission takes place in certain agricultural areas, while the urban cities may have another transmission during winter (December–February) due to broken water pipes [10]. The studies were conducted during the malaria transmission season in each site between 2010 and 2015.

### Study population

Patients were included if they met the following criteria: age 6 months or above, asexual parasitaemia of 500–100,000/µl, no mixed malaria infection, axillary temperature ≥37.5 °C or a history of fever within 24 h, ability to swallow medication, ability to report for follow-up, and informed consent. Patients were excluded if they had danger signs in children under 5 years of age, signs of severe malaria, concomitant febrile illness other than malaria, mixed infection or infection with another species, or were pregnant or lactating. Female minors from menarche to 18 years and unmarried women above 18 years were also excluded due to social and cultural norms related to requesting a pregnancy test from this population.

### Sample size

Estimation of the sample size in each site was based on an assumption of treatment failure of 5 %, with a 95 % confidence level and precision of 5 %. Therefore, the target sample size in each study was 88 cases. This was based on a calculated minimum sample size of 73 cases, with approximately 20 % more to allow for loss to follow-up and withdrawals during follow-up. In two AL studies in the towns of Gedaref and Sennar, the malaria transmission rates in 2013–14 and 2014–15 were too low and the intended sample size could only be achieved in two consecutive seasons. It was decided to analyse the cases from each season separately, although this meant a smaller sample size than originally intended in four studies.

### Ethical considerations

The studies presented were part of the Sudan NMCP surveillance of therapeutic efficacy of anti-malarial drugs. Permission to conduct these studies was obtained from the Federal Ministry of Health of Sudan and the WHO ethical review committee and community leaders. Prior to enrolment, individual informed consent was read to all study patients. Consent was requested from guardians of children aged less than 17 years. In addition, an assent form was signed by children aged 12–17 years old. If the patient or parent/guardian was illiterate, the study information was read to each patient/guardian in the presence of a witness known to the patient or a community leader.

### Treatment and follow-up

The studies were one-arm prospective studies, conducted according to the standard WHO protocol for monitoring therapeutic efficacy of anti-malarials against uncomplicated falciparum malaria for 28 days [5]. Treatment in the AL studies and in and the AS + SP studies was given according to the dose regimens and therapeutic ranges recommended for each drug by WHO guidelines [11]. AL and AS + SP tablets used in the studies were provided by the WHO.

### Rescue medication

Patients who developed danger signs and/or severe malaria were given parenteral artesunate, according to the national treatment policy. Treatment failures after AL were treated with quinine and treatment failures after AS + SP were re-treated with AL.

### Laboratory methods

Thick and thin blood films for malaria microscopy were prepared, stained with Giemsa and examined, as described by the WHO [12]. All blood smears were checked by a second microscopist and discordant results with differences in species or in parasite density of were re-read by a third microscopist, and the average of the two most concordant readings was taken.

### Genotyping at baseline and recurrent infections

Blood samples were collected on filter paper (Whatman^®^ 3MM, GE Healthcare UK Ltd. Buckinghamshire, England) at enrolment and at any visit where parasites were observed on and after day 7. Filter papers were dried and each sample was stored in a separate plastic bag containing silica gel. All filter papers were subsequently transferred to Naval Medical Research Unit (NAMRU-3) (through the WHO Eastern Mediterranean Regional Office) for analysis. DNA was extracted from blood spots using the QIAamp^®^ DNA Mini Kit (Qiagen, Hilden Germany) as described by the manufacturer. Parasite DNA was extracted from blood spots on day 0 and on the day of reappearance of asexual parasitaemia. Blood samples were tested by nested PCR for *msp1*, *msp2* (merozoite surface proteins) and *glurp* (glutamate-rich protein) as described [13]. Samples of recurrent parasitaemia from day 7 onwards were tested by PCR to distinguish between re-infection and recrudescence according the WHO definitions [13]. If only two of the three loci could be amplified in paired samples and the PCR fragments in the paired samples were completely different in at least one locus, then the recurrent parasitaemia is considered a new infection. If the paired samples have at least one identical band and this was found at both loci amplified the recurrent sample was considered a recrudescence, even if some alleles were missing or new alleles were observed in the recurrent sample. If only one locus could be amplified and this marker indicates recrudescence, with shared bands, the sample was defined as recrudescence. When it was not possible to distinguish recrudescence from new infection, the sample was classified as indeterminate (unknown).

### Genotyping for SP-resistance genes

Mutations conferring resistance to SP were detected by analysing extracted DNA from all day 0 samples to determine the mutations in the *dihydrofolate reductase* (*dhfr*) and *dihydropteroate synthase* (*dhps*) genes in four AS + SP studies performed in 2011. Both *dhfr* and *dhps* genes were amplified using nested PCR as described elsewhere [14]. PCR products were subjected to DNA sequencing using BigDye terminator chemistry (Applied Biosystems, Foster City, CA, USA) on a 3130 × l Genetic Analyzer (Applied Biosystems, Foster City, CA). DNA sequences were assembled and mutations were verified by the inspection of both forward and reverse strands using BioEdit version 7.2.5. [15]. DNA sequencing analysis of the *dhfr* fragment was carried out to detect mutations at codons 16, 51, 59, 108 and 164, whereas the *dhps* fragment was evaluated at codons 436, 437, 540, 581 and 613.

### Statistical analysis

Data were analysed with a computer program developed by the WHO [5]. This software included the classification of treatment outcomes according to WHO definitions, with and without PCR, using per-protocol and Kaplan–Meier survival analysis. Patients who were excluded or withdrew after enrolment were not included in the per protocol analysis of treatment outcomes. In the Kaplan–Meier survival analysis, all such cases were included until the day of withdrawal from the study. Gametocyte carrier rates were compared between the two treatment groups using the Chi square test and survival analysis. Person gametocytaemia week (PGW) rates were calculated for each treatment group as the total number of weeks in which blood slides positive for gametocytes in all patients during a two-week follow-up, divided by the total number of follow-up weeks and expressed per 1000 person-weeks [16]. The Chi square test was used to compare the prevalence rates of haplotypes among four different sites included in the 2011 survey and also to assess temporal changes in the prevalence of rates haplotypes in Gedaref between 2011 results and 2003 published data. Fisher’s exact test was used for observations less than five. Analysis of variance (ANOVA) was used to test for differences between baseline study population characteristics. Regression analysis was used to test association of treatment failure with patient variables. Statistical analysis was performed using SPSS ^®^ software version 22, SPSS Inc., Chicago, USA). For comparison of pairs of categorical variables Chi square test was performed using Epi-Info 7 (CDC, Atlanta, GA and the WHO, Geneva, Switzerland).

## Results

### Efficacy studies on AL

The characteristics of the study population in nine AL studies are shown in Table 1. One-way ANOVA showed that there were differences between the sites in the mean age of the patients (F = 4.322, *p* < 0.001), mean temperature at baseline (F = 5.010), *p* = 0.002) and asexual parasite density at baseline (F = 11.980), *p* < 0.001). Table 2 shows the treatment outcomes based on per-protocol analysis and the cumulative incidence of treatment success (Kaplan–Meier survival analysis) with or without PCR correction. The PCR-corrected cumulative incidence of treatment success ranged from 96.8 to 100 %. Except for two cases (one classified as ETF on day 2 and, therefore, not tested for parasitaemia on day 3, and the other case classified as ACPR), all patients had complete clearance of microscopic asexual parasitaemia on day 3. The dosages of AL taken by the treatment failure cases were checked, and they were all found to be within the therapeutic range [11]. Cases of AL treatment failure showed no remarkable epidemiological pattern.

**Table 1:** Demographic and clinical characteristics of patient in therapeutic efficacy studies to evaluate artemether-lumefantrine or artesunate + sulfadoxine-pyrimethamine in Sudan (2010–2015)

Drug	Site	Study years	N	Males		Age group						Temperature D0		Parasite count (/uL), D0	
						<5 years		5–14 years		≥15 years					
				N	(%)	N	(%)	N	(%)	N	(%)	Mean	(SD)	Geometric mean	Range
AS + SP	Kosti	2010	92	57	(62)	14	(15)	59	(64)	19	(21)	38.1	(1)	26,202	(5470–90,940)
	Sennar	2010	97	51	(53)	5	(5)	69	(71)	23	(24)	38.4	(0.9)	13,973	(1800–71,889)
	Gedaref	2011–12	100	53	(53)	17	(17)	52	(52)	31	(31)	39	(1.1)	11,725	(1600–129,167)
	Kassala	2011	75	44	(59)	24	(32)	17	(23)	34	(45)	38.1	(0.8)	5917	(1020–68,421)
	Kosti	2011	98	55	(56)	17	(17)	63	(64)	18	(18)	38.1	(1.1)	14,249	(3380–88,098)
	Sennar	2011	95	43	(44)	9	(10)	71	(75)	15	(16)	37.9	(1.2)	10,592	(1400–57,910)
	Kosti	2012–13	95	45	(47)	20	(21)	58	(61)	17	(18)	37.1	(1.1)	18,541	(1120–97,143)
	Damazin	2013–14	100	61	(61)	19	(19)	67	(67)	14	(14)	37.7	(1)	15,657	(1460–90,000)
	Sennnar	2013–14	88	57	(65)	9	(10)	76	(86)	3	(3)	37.1	(0.9)	8252	(1140–35,500)
	New Halfa	2013–14	80	40	(50)	14	(18)	25	(31)	41	(51)	38.6	(1.8)	12,798	(2123–116,666)
	Gedaref	2014–15	93	47	(51)	15	(16)	58	(62)	20	(22)	37.6	(0.9)	11,165	(1053–52,319)
AL	Damazin	2010	67	32	(48)	0	(0)	30	(45)	37	(55)	38.2	(0.6)	11,203	(1675–100,000)
	Kassala	2010	85	57	(67)	11	(13)	40	(47)	34	(40)	37.8	(1)	11,245	(1500–97,142)
	Kosti	2012–13	101	64	(63)	7	(7)	44	(44)	50	(50)	38.2	(0.9)	5339	(1000–45,756)
	Damazin	2013	98	54	(55)	11	(11)	64	(65)	23	(24)	38.1	(0.9)	12,095	(3020–70,961)
	Kassala	2013–14	84	53	(63)	12	(14)	32	(38)	40	(48)	38	(0.7)	16,226	(1100–90,000)
	Sennar	2013–14	42	22	(52)	5	(12)	26	(62)	11	(26)	38.5	(1.2)	18,695	(3280–93,939)
	Gedaref	2013–14	35	26	(74)	1	(3)	10	(29)	24	(69)	37.6	(0.9)	8524	(1060–75,000)
	Sennar	2014	41	22	(54)	8	(20)	25	(61)	8	(20)	37.8	(1.1)	10,827	(1040-78,110)
	Gedaref	2014–15	42	29	(69)	7	(17)	23	(55)	12	(29)	37	(1.1)	4741	(1300–14,240)

*SD* standard deviation

**Table 2:** Parasitological and clinical outcomes in therapeutic efficacy studies to evaluate artemether-lumefantrine or artesunate + sulfadoxine-pyrimethamine in Sudan (2010–2015)

	Site	Study years	N	PD3	PCR un-corrected					PCR corrected						
					Excl/loss	ETF	LCF	LPF	ACPR	Excl/loss	ETF	LCF	LPF	ACPR	Cure rate (KM)	(95 % CI)
AS + SP	Kosti	2010	92	0	6	0	4	0	82	6	0	4	0	82	95.3	(88.1–98.2)
	Sennar	2010	97	0	5	0	4	1	87	6	0	3	1	87	95.7	(89.0–98.4)
	Gedaref	2011-12	100	0	0	0	3	10	87	4	0	3	6	87	91	(83.4–95.2)
	Kassala	2011	75	0	1	0	2	1	71	3	0	1	0	71	98.6	(90.7–99.8)
	Kosti	2011	98	0	8	0	5	0	85	13	0	0	0	85	100	(95.8–100)
	Sennar^a^	2011	95	0	1	0	6	7	81	12	0	0	1	81	98.9	(92.5–99.8)
	Kosti^a^	2012-13	95	0	7	0	1	4	83	11	0	0	0	83	100	(95.7–100)
	Damazin^a^	2013-14	100	0	26	0	3	5	66	30	0	1	2	66	95.9	(87.9–98.7)
	Sennnar	2013-14	88	0	0	0	2	6	80	5	0	1	2	80	96.4	(89.2–98.8)
	New Halfa	2013-14	80	0	0	1	0	0	79	0	1	0	0	79	98.7	(91.4–99.8)
	Gedaref^a^	2014-15	93	1	0	0	7	15	70	10	0	2	10	70	86.5	(77.5–92.1)
AL	Damazin	2010	67	0	0	1	0	0	66	0	1	0	0	66	98.5	(89.9–99.8)
	Kassala	2010	85	0	1	0	0	0	84	1	0	0	0	84	100	(95.7–100)
	Kosti	2012-13	101	0	10	0	1	1	89	12	0	0	0	89	100	(95.9–100)
	Damazin	2013	98	0	12	0	1	6	79	18	0	0	1	79	98.9	(92.1–99.8)
	Kassala^a^	2013-14	84	0	1	0	1	0	82	1	0	0	0	82	100	(95.6–100)
	Sennar-Almazad	2013-14	42	0	11	0	0	2	29	12	0	0	1	29	96.8	(79.2–99.5)
	Gedaref-Rawashda	2013-14	35	0	0	0	0	0	35	0	0	0	0	35	100	n/a
	Sennar-Abei	2014	41	0	0	0	1	1	39	1	0	1	0	39	97.5	(83.5–99.6)
	Gedaref-Karari	2014-15	42	1	0	0	0	1	41	0	0	0	1	41	97.7	(84.3–99.7)

*PD3* positive on day 3, *LCF* late clinical failure, *LPF* late parasitological failure, *ACPR* adequate clinical and parasitological response, *KM* Kaplan–Meier, *CI* confidence interval

^a^In the PCR-corrected analysis, one patient was excluded, due to unknown PCR in AS + SP studies in Sennar (2011), Kosti (2012–13), Damazin (2013–14), Gedaref (2014–15) and in AL Kassala study of 2013–14

### Efficacy studies on AS + SP

Eleven therapeutic efficacy tests were conducted to evaluate AS + SP efficacy. The characteristics in the study population in each site are shown in Table 1. ANOVA showed no significant difference in gender (F = 1.692, *p* = 0.078), but there were significant differences in the mean age (F = 5.852, *p* < 0.001), the mean body temperature at baseline (F = 21.030, *p* < 0.001) and the asexual parasite density at enrolment (F = 15.936, *p* = 0.001).

The lowest PCR-corrected cure rates in the AS + SP studies were reported in two studies in Gedaref (Table 2). The first study, performed in 2011-12, reported a cure rate of 91.0 % (95 % CI 83.4–95.2). The second study, performed in the 2014-15 season, reported a cure rate of 86.5 % (95 % CI 77.5–92.1). In the other studies, the cure rates of AS + SP ranged between 95.6 and 100 %. The dosages of AS + SP taken by treatment failure cases were checked and they were confirmed to be within the recommended therapeutic range [11].

When tested by binary logistic regression, the PCR-corrected treatment failure had no significant association with the baseline characteristics of gender, body temperature and parasite density. However, there was significant association with age of the patient (Wald Chi square = 10.062, *p* = 0.002). The rate of treatment failure in Gedaref was significantly higher than all other studies (21/179 = 11.7 % versus 17/731 = 2.3 %), Chi square = 31.794, OR 5.582 (95 % CI 2.879–10.825). In the two Gedaref studies, the rate of treatment failure in children under 5 was significantly higher than in adults (≥15 years), (8/28 = 28.6 % versus 2/51 = 3.9 %, Fisher’s exact test, *p* = 0.003).

### Gametocytaemia after treatment

**Fig. 2:**
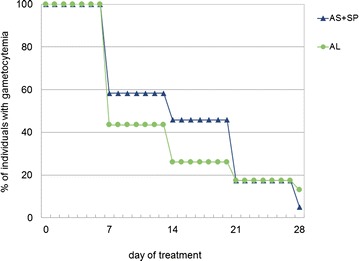
Time to disappearance of microscopic gametocytaemia in gametocyte-positive individuals at enrolment and following treatment. *AL* artemether-lumefantrine (n 23), *AS* *+* *SP* sulfadoxine-pyrimethamine (n 24)

The baseline prevalence of gametocytaemia in the two treatment groups was not significantly different: 2.4 % in the ASSP group and 3.9 % in the AL group (Chi square = 3.999, *p* = 0.083, OR 1.663, 95 % CI 0.930–2.973). Figure 2 is a Kaplan–Meier plot showing the time to clearance of gametocytes in gametocyte-positive individuals by microscopy at enrolment and during follow-up. In the AL group, there was a reduction of 56.5 % in the proportion of patients with gametocytaemia by day 7. The reduction from baseline by days 14, 21 and 28 were 73.9, 82.6, and 87.0 %, respectively. The proportions of patients with gametocytaemia in the two treatment groups were not significantly different on days 7, 14, 21 and 28. Of those who were gametocyte-negative at baseline, there was no significant difference in the proportion of patients who developed gametocytaemia during follow-up in the AL group (4/572, 0.9 %) compared to the ASSP group (16/958, 1.7 %), Fisher’s exact test, *p* = 0.242. There was no significant difference between the two treatment groups in the gametocyte carriage rates, measured as gametocytaemia per 1000 person-weeks, 17.7 with AL and 20.2 with ASSP, RR 0.89, 95 % CI 0.62–1.28, *p* = 0.53.

### Comparison of molecular markers of SP resistance among four sites in 2011

Molecular markers of SP resistance were studied in samples collected in 2011 from four sites: Kassala, Gedaref, Kosti and Sennar. Genetic mutations associated with SP resistance in *dhfr* and *dhps* were successfully analysed in 365 and 366 samples, respectively, and mutation combinations were identified in both genes in 361 samples.

Table 3 shows an analysis of the mutations detected in the *dhfr* gene in four sites. Gedaref had a significantly higher frequency of the *dhfr* triple mutant N51I + C59R + S108 N than Kassala, Kosti and Sennar. Table 4 shows an analysis of the haplotypes in the *dhps* gene. The frequency of the wild-type *dhps* haplotype differed by site, and it was significantly higher in Kosti than in Gedaref, Kassala and Sennar. The dominant resistant *dhps* haplotype was the double mutant A437G + K540E. The *dhps* triple mutant haplotype A437G + K540E + A581G, which predominated in 69 samples, was detected only in one sample in Kosti but in 40.7 % of samples from Kassala, 15.6 % in Gedaref and 20 % in Sennar.

**Table 3:** Comparison of the frequency of *dhfr* gene haplotypes in four sentinel sites in 2011

Number of mutations	Mutation haplotype	KASSALA N = 79	GEDAREF N = 96	SENNAR N = 98	KOSTI N = 92	p value*
None	*dhfr* wild type	2 (2.5 %)	2 (2.1 %)	4 (4.1 %)	7 (7.6 %)	0.27
Double	N51I, S108 N	73 (92.4 %)	67 (69.8 %)	87 (88.8 %)	78 (84.9 %)	<0.001
	N51I, C59R	0	1 (1.0 %)	0	1 (1.0 %)	
Triple	N51I, C59R, S108 N	4 (5.1 %)	26** (27.1 %)	7 (7.1)	6 (6.5 %)	<0.001

* Fisher’s exact test

** Significantly higher than Kassala (Fisher’s exact *p* = 0.0001), Kosti (Fisher’s exact *p* = 0.0002) and Sennar (Fisher’s exact *p* = 0.0002)

**Table 4:** Comparison of the frequency of *dhps* gene haplotypes in four sentinel sites in Sudan 2011

Number of mutations	*Dhps* haplotype	Kassala	Gedaref	Sennar	KostiI	Pearson’s Chi square	p value
None	wild *dhps* type	14 (17.3 %)	22 (22.9 %)	30 (30.0 %)	57(64.0 %)^a^	52.127	<0.001
Single	S436A	0	2	1	2		
	A437G	0	4	0	4		
	K540E	0	3	1	0		
	A581G	1	0	1	1		
	S436C	0	0	9	0		
Double	A437G, K540E	26 (32.1 %)	44 (45.8 %)	34 (34.0 %)	24 (27.0 %)^b^	7.824	0.050
	S436C, A581G	0	0	2	0		
	S436C, K540E	0	0	1	0		
	A437G, A581G	0	0	1	0		
Triple	S436A, A437G, K540E	6 (7.4 %)	6 (6.2 %)	0	0		
	A437G, K540E, A581G	33 (40.7 %)	15 (15.6 %)	20 (20 %)	1 (1 %)^c^		<0.001^d^
Quadruple	S436A, A437G, K540E, A581G	1	0	0	0		
Total		81	96	100	89		

^a^Kosti wild type is significantly higher than Gedaref (Chi square = 31.93, *p* < 0.001), Kassala (Chi Square = 38.12, *p* < 0.001) and Sennar (Chi square = 21.97, *p* < 0.001)

^b^Kosti mutant is significantly lower than Gedaref (Chi square = 7.07, *p* = 0.008), not significantly different from Kassala (Chi Square = 0.54 *p* = 0.462) or Sennar (Chi square = 1.10*, p* = 0.295)

^c^The proportion of Kosti mutant isolates is significantly lower than Gedaref p < 0.001, Kassala, *p* < 0.001 and Sennar *p* < 0.001, Fisher’s exact test

^d^Fisher’s exact test

Table 5 shows the distribution of mutations for the two-locus haplotypes of the combined *dhfr* and *dhps* genes. The most frequent mutation combinations were quadruple mutations (n = 111) followed by double mutations (n = 97) and quintuple mutations (n = 87). The dominant haplotype was the quadruple mutation N51I + S108 N/A437G + K540E (n = 106). The proportion of isolates with quintuple or sextuple haplotypes was highest in Kassala (38/79 = 48.1 %). This was not significantly higher than in Gedaref ([37/96 = 38.5 %], Chi square = 1.617, *p* = 0.2035), but it was significantly higher than in Kosti ([4/92 = 4.4 %], Fisher’s exact test, *p* < 0.001) and in Sennar [(18/98 = 18.4 %), Fisher’s exact test, p < 0.001]. The proportion of isolates with quintuple or sextuple mutations in each of the four sites studied is shown in the map indicating the geographical location of the sites (Fig. 1). Among the analysed isolates collected for four sites in 2011, there were nine treatment failures in whom the two-locus genotyping was successful. This number was too small for a meaningful analysis of the association between treatment failure and molecular markers.

**Table 5:** The types and frequencies of SP resistance haplotypes detected in both *dhfr* and *dhps* genes in isolates from four sentinel sites in Sudan in 2011

Haplotypes	Kassala, n = 79	Gedaref, n = 96	Sennar, n = 98	Kosti, n = 88	Total	p value*
	N (%)	N (%)	N (%)	N (%)	N	
*dhfr wild type* + dhps wild type	1 (1.3)	1 (1)	4 (4.1)	6 (6.8)	12	0.133
All double mutants	9 (11.4)	17 (17.7)	23 (23.5)	48 (54.5)	97	<0.001
*dhfr* N51I, S108 N + *dhps* wild type	9 (11.4)	17 (17.7)	23 (23.5)	47 (53.4)	96	<0.001
*dhfr* wild type + *dhps* A437G + K540E	0 (0)	0 (0)	0 (0)	1 (1.1)	1	
All triple mutants	6 (7.6)	13 (13.5)	15 (15.3)	11 (12.5)	45	0.467
*dhfr* N51I, C59R, S108 N + *dhps* wild type	4 (5.1)	4 (4.2)	3 (3.1)	4 (4.5)	15	0.911
*dhfr* N51I, S108 N + *dhps* S436A	0 (0)	0 (0)	1 (1.0)	2 (2.3)	3	
*dhfr* C59R, S108 N + *dhps* S436A	0 (0)	1 (1)	0 (0)	0 (0)	1	
*dhfr* N51I, S108 N + *dhps* A437G	0 (0)	4 (4.2)	0 (0)	4 (4.5)	8	
*dhfr* N51I, S108 N + *dhps* K540E	0 (0)	3 (3.1)	1 (1.0)	0 (0)	4	
*dhfr* N51I, S108 N + *dhps* A581G	1 (1.3)	0 (0)	1 (1.0)	1 (1.1)	3	
*dhps* A437G, K540E, A581G	1 (1.3)	1 (1)	0 (0)	0 (0)	2	
*dhfr* N51I, S108 N + *dhps* S436C	0 (0)	0 (0)	9 (9.2)	0 (0)	9	
All quadruple mutants	25 (31.6)	28 (29.2)	38 (38.8)	20 (22.7)	111	0.125
*dhfr* N51I, C59R, S108 N + *dhps* S436A	0 (0)	1 (1)	0 (0)	0 (0)	1	
*dhfr* N51I, S108 N + *dhps* A437G, K540E	25 (31.6)	27 (28.1)	34 (34.7)	20 (22.7)	106	0.325
*dhfr* N51I, S108 N + *dhps* A437G, A581G	0 (0)	0 (0)	1 (1.0)	0 (0)	1	
*dhfr* N51I, S108 N + *dhps* A436C, K540E	0 (0)	0 (0)	1 (1.0)	0 (0)	1	
*dhfr* N51I, S108 N + *dhps* A436C, A581G	0 (0)	0 (0)	2 (2.0)	0 (0)	2	
All quintuple mutants	37 (46.8)	33 (34.4)	14 (14.3)	3 (3.4)	87	<0.001
*dhfr* N51I, C59R, S108 N + *dhps* A437G, K540E	0 (0)	17 (17.7)	0 (0)	2 (2.3)	19	<0.001
*dhfr* N51I, S108 N + *dhps* S436A, A437G, K540E	5 (6.3)	5 (5.2)	0 (0)	0 (0)	10	0.004
*dhfr* N51I, S108 N + *dhps* A437G, K540E, A581G	32 (40.5)	11 (11.5)	14 (14.3)	1 (1.1)	58	<0.001
All sextuple mutants	1 (1.3)	4 (4.2)	4 (4.1)	0 (0)	9	
*dhfr* N51I, C59R, S108 N + *dhps* S436A, A437G, K540E	0 (0)	1 (1)	0 (0)	0 (0)	1	
*dhfr* N51I, C59R, S108 N + *dhps* A437G, K540E, A581G	0 (0)	3 (3.1)	4 (4.1)	0 (0)	7	
*dhfr* N51I, S108 N + *dhps* S436A, A437G, K540E, A581G	1 (1.3)	0 (0)	0 (0)	0 (0)	1	

* Associations tested for the main groups, using the Chi square test, Fisher’s exact test was used when the number of observations was less than five

### Comparison of molecular markers from Gedaref in 2003 and 20011

To assess the changes over time in the *dhfr* and *dhps* genotypes in Gedaref, comparisons were made with published reports by Al-Saai et al., who studied isolates collected in 2003 from Asar village, which located approximately 16 kilometres south of Gedaref town [17, 18], and with findings reported for isolates collected in 2003 from the two villages of Daraweesh and Kajara located a few hundred meters apart at a distance of 15 km south of Gedaref town (locations indicated in the map, Fig. 1) [19]. For the sake of assessing temporal changes, we assume that all of these neighbouring localities represent the Gedaref site. The most notable change was observed in the triple mutant N51I/C59R/S108 N which was absent [17] or observed in very low frequency of 0.6 % [19] in 2003 samples but in 2011, it escalated to 27.1 %. There is also a significant displacement of the *dhfr* wild-type haplotype (from 10.6 % in 2003 [17] to 2.1 % in 2011, *p* = 0.018, Fisher’s exact test). In the *dhps* gene there is emergence of the point mutation S436A, which was absent in the two cited surveys performed in 2003 [17, 19]. Other significant changes are related to combination of mutations in the *dhps* with the *dhfr* genes. The quintuple mutant N51I/C59R/S108 N + A437G/K540E, which was reported in very low frequencies in 2003 isolates from Gedaref (1/146, 0.7 %) [19], increased to 27.1 % (26/96) in the 2011 sample from Gedaref (*p* < 0.001, Fisher’s exact test). Additionally, the proportion of isolates with quintuple/sextuple mutations in Gedaref has increased from 13.1 % in 2003 [19] to 38.6 % in 2011 (Fisher’s exact test, *p* < 0.001).

## Discussion

The high cure rate of AL in the present study was consistent with reports from other endemic areas [20, 21]. The proportion of patients who are parasitaemic on day 3 of treatment is the indicator used during routine monitoring to identify suspected artemisinin resistance in *P. falciparum* [22]. With one exception, all patients treated with AL were negative for asexual parasitaemia on day 3, indicating that artemether effectively reduced the parasite biomass [23]. Artemisinin resistance, defined as a delayed parasite clearance time, has so far been reported only in Southeast Asia [24, 25]. A clear distinction must be made between treatment failure, i.e., the absence of resolution of parasitaemia and clinical signs after anti-malarial treatment, and true resistance to an anti-malarial drug [26]. The outcome of a therapeutic efficacy test is influenced by a triad of the patient’s immunity, the parasite’s susceptibility to the drug and pharmacokinetics, which may vary from person to person. Lumefantrine is highly lipophilic, and its bioavailability depends on concurrent food intake, which could be impaired during acute malaria [27]. The definitive confirmation of resistance to an anti-malarial drug requires proof that the parasites are recrudescent and demonstration that an effective blood concentration of the drug or its metabolites has been maintained for at least four parasitic cycles (approximately 6 days) [28]. Pharmacokinetic studies required to provide such evidence are not usually performed in standard therapeutic efficacy studies aimed at helping policy makers to make decisions regarding the selection of first-line and second-line treatments for uncomplicated malaria [5]. In the absence of pharmacokinetic data, the causes of the treatment failure in the present study may only be speculated upon and it could be due to true drug resistance or poor bioavailability of the drugs.

The PCR-corrected cure rates of AS + SP in Gedaref in 2014–15 were 86.5 %, below the WHO threshold for changes in treatment policy [11]. In all other sites tested, however, AS + SP was efficacious, with cure rates ranging from 95.6 to 100 %. In contrast to the present study conducted in Kassala in 2011, which found a cure rate of 98.6 %, a subsequent study in 2012 in the same site reported a PCR-corrected per-protocol cure rate of 93.7 % with AS + SP [29]. This trend indicates a possible decline in treatment efficacy. Apart from the obvious disadvantages to the patient being treated with an ineffective drug, continued use of an ACT in which the partner drug is failing would compromise the efficacy of the artemisinin component and expose the parasites to selection pressure for artemisinin resistance. No asexual parasites were detected on day 3 by microscopy, indicating that the artesunate component had achieved its expected initial rapid reduction in the parasite biomass [23]. Patients who failed treatment likely had submicroscopic levels of parasitaemia on day 3. Therefore, SP failed to clear the residual parasites, leading to recrudescence. This was also supported by the fact that almost all of the treatment failures were late treatment failures (37/38). The AS + SP treatment failure in Gedaref in the 2014–15 season surpassed the failure rate of 10 % recommended by the WHO for policy change and, therefore, underscores the need to update the malaria treatment policy in Sudan.

In the present findings, the risk of treatment failure was clearly much higher in children who are less than 5 years of age compared to adults. The much higher rate of treatment failure in young children could be associated with age-related immunity. Theander [30] reported that the outcome of malaria infection in this area is strongly associated with age-related immunity. Djimdé et al. [31] found that the ability to clear chloroquine-resistant parasites was strongly associated with age, which they regard as the most consistent correlate of protective immunity in areas endemic for *P. falciparum* malaria. In the present study, the risk of treatment failure was clearly much higher in children less than 5 years of age compared to adults. It is also notable that the age composition in the samples from the different sites shows wide variations. This means that the age composition of the study sample in this area could influence the overall results of a trial, a fact that should be taken into consideration in the planning and analysis of trials in such a setting. Because the blood levels of the drugs have not been measured in the present study, the possible impact of pharmacokinetic factors that might contribute to the increased risk of SP treatment failure in young children cannot be ruled out [32].

Few studies have been performed to compare the impact of AL and AS + SP on gametocytaemia [33]. In the present study, there was no significant difference in the clearance of microscopic gametocytaemia between AL and AS + SP. This is in line with a trial reported by van den Boek et al. [34], which compared the efficacy of three artemisinin-based combinations, AS + SP, AL and artesunate +amodiaquine (AS + AQ). They found no significant differences between these drugs on gametocytaemia by day 28. However, another study in Yemen reported a significantly higher rate of gametocyte clearance with AL compared to AS + SP [35]. The present study also showed no significant difference between the two drugs in their effect on gametocyte carriage as measured by PGW.

The two main factors in the spread of anti-malarial drug resistance are drug pressure and human migration [36]. Because of its long elimination half-life, SP is particularly vulnerable for the induction of resistance by drug pressure [37]. Drug pressure is an important factor in Sudan, where self-medication and sub dosing with anti-malarial drugs is common [38–40]. It is not surprising that the failure of AS + SP first emerged in Gedaref or Eastern Sudan in close proximity to the borders with Ethiopia. This area has historically been a hotbed for drug-resistant malaria. Chloroquine resistance was first reported in the eastern Sudan, particularly in the refugee camps [41, 42]. The highest rate of treatment failure of SP as a monotherapy has also been reported in Gedaref [4]. Mass population movements take place in this region and camps hosted refugees from Eritrea and Ethiopia for decades, in addition to the continuous exchange of populations with Ethiopia and Eritrea for economic reasons. SP was introduced as a first-line treatment for falciparum malaria in Ethiopia in 1998–1999, but within a few years, the mean SP treatment failure rates reached 35.9 and 71.8 % for day 14 and day 28, respectively [43]. Population movements are believed to play an important role in the epidemiology of anti-malarial drug resistance in Africa [44]. The temporal and regional changes in drug resistance observed in the present study highlight the need for further studies to understand the exact contribution of population movements and other demographic factors in the emergence and spread of drug resistance in this area.

A number of mutations in the *dhfr* and *dhps* genes have been associated with SP resistance [6, 45–48]. In Africa, SP resistance is strongly associated with combinations of five single point mutations, *dhfr* N51I, C59R, S108 N, and *dhps* A437G and K540E [49–52]. The prevalence of the different antifolate markers in the four sites studied in the present study indicates varying degrees of selection for resistant haplotypes in the different sites. The emergence and increased frequency of the C59R mutation in the four sites tested in the present study is particularly interesting. In contrast to the situation in other parts of Africa, the *dhfr* triple mutation N51I, C59R, S108 N has maintained a low frequency in eastern Sudan because of the low prevalence of the C59R mutation. The C59R mutant was reported to be absent in eastern Sudan isolates in 2003 [17, 53], and it was found at a very low frequency of 0.6 % in another study [19]. After analysis of isolates collected in 2007, Menegon et al. also reported the absence of the C59R mutation in samples from Gedaref, but it was present in isolates from Gezira in central Sudan [54]. Gadalla et al. [29] reported a low prevalence (1/59) of this mutant in isolates collected in Kassala in 2012. Longitudinal studies in eastern Sudan and in Gedaref have indicated that the prevalence of the S108 N mutation has gradually increased since 1990 [55]. In 2012, isolates from Kassala, the prevalence of the *dhfr* double mutant N51I + S108 N reached 100 %, with a rare triple mutant N51I + C59R + S108 N haplotype in one individual [29]. In the present study, the increase in the prevalence of the triple mutation *dhfr* I51/R59/N108 is associated with displacement of the *dhfr* double mutant in Gedaref, indicating that the triple mutant has emerged in the background of a double mutant *dhfr,* which has previously maintained a high prevalence level in this area as well as in locations in Sudan.

Mutations in the *dhps* gene, which confer resistance to the sulfadoxine component of SP, appeared in Africa in 1990s [52]. In eastern Sudan, the *dhps* double mutant A437G + K540E was absent in Gedaref (Asar village) in 1993; it appeared in 1998 and increased dramatically in 2000 following the use of SP as a first-line treatment for malaria [55]. An interesting finding in the present study was that for the first time in Sennar, mutant S436C was detected in 12 isolates. Malisa et al. also reported this mutant at a low frequency for the first time in Tanzania, also in the context of SP resistance and drug selection pressure [56]. They attributed their ability to detect this rare mutant to a larger sample size used and the inability of alternative methodologies to detect a rare codon 436 mutation. However, the frequency of this mutant in our Sennar sample was not rare (12 %). Moreover, the mutant was not detectable in the other three sites assessed together in our study. This could possibly be another manifestation of selection pressure. The present study detected other changes in the *dhps* suggestive of selection for resistant haplotypes, including the emergence of the triple mutant S436A + A437G + K540E. Apart from Gedaref, the cure rates were still high in the other three sentinel sites, but the high prevalence of quintuple mutations in Kassala and Gedaref and the temporal changes in the frequency of these markers in Gedaref should be cause for concern about the emergence and spread of SP resistance on a wider scale within the country.

The present study shows significant regional differentiation in the SP molecular markers in the four locations covered by the molecular markers survey. In contrast to the high prevalence of quintuple mutants found in Kassala and Gedaref, which are close to the Ethiopia and Eritrea borders, the majority of samples from Kosti are triple mutants or less. This differentiation is probably due to interplay of different demographic and genetic factors as well as the effect of drug pressure and the influence of human migration on the dispersal of resistant parasites. In Ethiopia, the prevalence of molecular mutations associated with SP resistance is high, reaching saturation levels [57, 58]. Pearce et al. [44] used the microsatellite polymorphism flanking the *dhps* gene to determine which resistance alleles shared common ancestry and found that *dhps* alleles from Ethiopia and eastern Sudan have a distinct lineage that is unique to the region. This lineage is believed to have emerged in Ethiopia and Sudan at the same time.

Molecular markers could constitute a powerful tool for the surveillance of SP resistance. By following the changes in SP resistance markers, it could be possible to follow the dispersal of resistant parasites and identify regions of significant parasite exchange in real time. This is particularly important in areas where molecular markers have been suggested as tools to track the importation of resistant parasites into malaria elimination areas [59]. There is a need high for resolution mapping of parasite genetics to be able to detect changes in drug resistance genes. In this regard, the importance of studying the regional variations in the frequencies of molecular markers within these countries should be highlighted. Some published reports have taken regional data as a national baseline to assess changes in other regions of the country. The regional differences shown in the present study, even among locations within Eastern Sudan, demonstrate that such an approach could lead to erroneous conclusions. The distance between Gedaref and Kassala is 200 km. However, the frequency of the *dhfr* 59R mutant is 5.1 % in Kassala and 28.1 % in Gedaref. The frequency of A436C is 12 % in Sennar and 0 % in all of the other sites. The present study also underscores the importance of collaboration between neighbouring endemic countries in the surveillance of drug resistance and the formulation of drug policies.

## Conclusions

Of particular concern is the finding in Gedaref, where the therapeutic efficacy of AS + SP is below the level recommended for treatment policy change, combined with a high prevalence of quintuple or sextuple *dhfr/dhps* mutations. Similary in Kassala, a high prevalence of quintuple or sextuple haplotypes was observed. This underscores the need to begin to review the national treatment policy. The finding of a high efficacy of artemether-lumefantrine in the study areas supports its use as a safe and effective second-line treatment against uncomplicated falciparum malaria in Sudan, in line with the current treatment policy. The survey of molecular markers for SP resistance showed regional differentiation and the selection of SP resistance haplotypes. This regional differentiation should be taken into account in the analysis of countrywide data. This study highlights the significance of considering human migration in the spread of drug resistance and the value of molecular marker studies in tracking the dispersal of resistant parasites.

## Abbreviations

AL: artemether–lumefantrine
SP: sulfadoxine-pyrimethamine
ACT: artemisinin-based combination therapy
AS + SP: artesunate-sulfadoxine-pyrimethamine
ETF: early treatment failure
LCF: late clinical failure
LPF: late parasitological failure
ACPR: adequate clinical and parasitological response
*dhfr*: dihydrofolate reductase gene
*dhps*: dihydropteroate synthase gene
PGW: person gametocyte-week
